# Understanding the value of biobank attributes to researchers using a conjoint experiment

**DOI:** 10.1038/s41598-023-49394-6

**Published:** 2023-12-20

**Authors:** Deepshikha Batheja, Srishti Goel, Warren Fransman, Anna Mantsoki, Stefano Ongarello, Ramanan Laxminarayan

**Affiliations:** 1 One Health Trust, Obeya Pulse, First Floor, 7/1, Halasur Road, Bengaluru, Karnataka 560102 India; 2 Specimen Bank, FIND, Geneva, Switzerland; 3 FIND, Geneva, Switzerland

**Keywords:** Biomarkers, Health care, Medical research

## Abstract

Biobanks are important in biomedical and public health research, and future healthcare research relies on their strength and capacity. However, there are financial challenges related to the operation of commercial biobanks and concerns around the commercialization of biobanks. Non-commercial biobanks depend on grant funding to operate and could be valuable to researchers if they can enable access to quality specimens at lower costs. The objective of this study is to estimate the value of specific biobank attributes. We used a rating-based conjoint experiment approach to study how researchers valued handling fee, access, quality, characterization, breadth of consent, access to key endemics, and time taken to fulfil requests. We found that researchers placed the greatest relative importance on the quality of specimens (26%), followed by the characterization of specimens (21%). Researchers with prior experience purchasing biological samples also valued access to key endemic in-country sites (11.6%) and low handling fees (5.5%) in biobanks.

## Introduction

Biobanking involves the collecting, processing, and storing of human biospecimens and relevant personal and health information for research purposes^1–8^. Biobanks predominantly provide value to their users for biomedical and health research in academic, non-commercial, and commercial product research and development. Biological samples are typically linked to background patient data including health records, family history, lifestyle, and genetic information. They are also referred to as biological specimens, biospecimens, or bio-samples.

Biobanks can provide researchers with access to a large and diverse range of biological samples that are necessary for drug discovery and development^9^. These bio-samples can be used to identify potential drug targets, test the efficacy and safety of drugs, basic and translational research, and validate diagnostic tests. They help accelerate the drug development process by reducing the time and cost required for clinical trials. By providing access to large numbers of well-characterized patient bio-samples, biobanks can help researchers identify patient subpopulations that may respond differently to a particular drug, enabling more targeted clinical trials. Biobanks can facilitate the development of personalized medicine by enabling identification of genetic, molecular, and other biomarkers that predict an individual’s response to a particular drug.

Despite their significant value in support of research, biobanks are not always financially sustainable. Sustainability challenges could force biobanks to store biospecimens of lower quality or destroy bio-samples over time because of the cost of storage^7,10^. These impediments are faced particularly by non-profit biobanks because they rely heavily on funding from external sources and often charge researchers only a handling fee, including retrieval and shipping of biospecimens, as a cost^11^. They do not charge the costs related to operating and maintaining the biobank. Private biobanks with cost recovery strategies have emerged to enhance the sustainability and utility of biobanks^12–14^. However, the commercialization of biobanks, motivated by profit, private interest, economic benefit and achieving financial viability goals, has raised questions about the ethical and legal implications of biobanking^14–17^. Due to these reasons, commercial biobanks may not be able to provide access to quality and well-characterized specimens, particularly from rare diseases or endemic sites^18^. This places limits on both future research and the goal to promote health equity for all. Therefore, understanding the value of various attributes of biobanks to researchers is pertinent in strengthening research capacity for biomedical and public health research^19–21^.

Here, we report the results of a conjoint analysis assessing the incremental economic value of attributes of biobanks that could improve their value for researchers. A conjoint analysis methodology assumes that individuals decide on preferences for goods and services based on a collection of features/characteristics rather than only one^22–25^. A key assumption in this type of analysis is that any product can be broken down into a set of attributes that eventually impact users’ perceived value of the product. So, each decision by the user involves assessing all the features/characteristics of these attributes, conducting value trade-offs on hypothetical product profiles, and finally choosing a product that represents their preferences. For instance, in this case, when assessing whether researchers were concerned about the handling cost of a bio-sample, we studied how researchers value handling costs relative to other attributes or characteristics associated with biobanks. The attributes of biobanks that we studied were handling fees, breadth of consent, time taken to fulfil requests, availability of a variety of specimens, confidence in the quality of specimens, well-characterized specimens, and access to key endemic in-country sites. Each attribute had two levels, and the research participants ranked randomly generated combinations of these biobank attributes and their levels, forming various biobank options.

## Material and methods

### Data

We surveyed experts in the biomedical and health research fields from 25 countries to evaluate the baseline value of biobanking services. We employed the convenience sampling method to identify: (1) clinical development leaders at diagnostic companies; (2) biomedical research universities and health institutes; and (3) pharmaceutical companies dealing with biomedical research from our network to be part of this study. In total, we surveyed 78 individuals between May and October 2022, using unique online survey forms based on the random combination of attributes and levels forming biobank profiles. The survey also included questions on respondent characteristics such as education, work experience, and experience with biobanks.

After the background questions, each respondent was asked to rate 16 biobank profiles (including all seven attributes) on a scale of 1–10. For this, the levels were randomized for each attribute to create various sets of biobank profiles. The attributes and levels of biobanks covered in this study have been detailed in Table 1. The levels indicated as “HIGH” were coded as “1” and those indicated as “LOW” were coded as “0” in the regression analysis. For each respondent, 16 biobank profiles were randomly generated using STATA, then from these 16 biobank profiles, groups of four were randomly generated to form choice sets of four biobank profiles to appear on the survey at once. This was done because respondents cannot rank 16 biobank profiles at once, so we created four choice sets of four biobank profiles each to appear on their screen at any given time.

**Table 1:** Conjoint experiment design summary: attributes and levels of biobanks.

S. no.	Attributes	Levels	Definition of attributes
1	Handling fee (high handling fee)	More than $50 per bio-sample (HIGH) Less than $50 per bio-sample (LOW)	Handling fee refers to the costs related to retrieval and shipping of bio-sample
2	Breadth of consent	Blanket (HIGH), Specific (LOW)	Breadth of consent includes (i) specific consent which indicates that bio-samples may be used only for the specific research that the bio-sample was requested for and, (ii) blanket consent, which indicates that bio-samples can be used for a wide array of secondary research
3	Time taken to fulfil bio-sample requests (timely fulfilment)	Within 3 months (HIGH), Longer than 3 months but within 6 months (LOW)	This is indicative of timely fulfilment of bio-sample requests, which refers to the time taken between the request and the delivery of the bio-sample
4	Availability of a variety of bio-sample types	Requested bio-sample type always available (HIGH) Limited bio-sample types available (LOW)	Availability of a variety of bio-sample types refers to the how easily an array of bio-samples may be available
5	Access to key endemic in country sites	Easily accessible (HIGH), Not easily accessible (LOW)	Access to key endemic in country sites refers to the ability to access bio-samples of diseases that are endemic to specific countries or regions, where the respondent may or may not be residing
6	Confidence in the quality of bio-sample (quality of specimen)	Had confidence in the quality of bio-sample (HIGH), Bio-sample quality was questionable (LOW)	Confidence in the quality of a bio-sample refers to the degree to which one can be certain that the sample is accurate and representative of the biological source from which it was obtained
7	Characterization of biospecimen (Well-characterized specimens)	Well-characterized biospecimens available (HIGH), Little to no bio-sample characterization (LOW)	Characterization of a biospecimen refers to the process of identifying and describing its biological properties

The primary outcome variable in this study is a measure of each respondent’s intention to use any of the 16 biobank profile options provided to them. These biobank profile options were randomly assigned and appeared in sets of four on their screen (labelled 1–4 in the first choice set, 5–8 in the second choice set, 9–12 in the third choice set, and 13–16 in the fourth choice set). The respondents were asked to rank each biobank profile option on a ten-point numeric scale from 1 (“not likely at all”) to 10 (“very likely”).

### Conjoint experimental design and analytical strategy

The survey used a conjoint analysis, which is a survey-based experiment method. This type of analysis is widely used in market, political science, and health economics research to determine how people value attributes of various alternatives when making choices involving trade-offs^22–25^. Specifically, we used a rating-based conjoint analysis method where respondents provide a numerical rating representing their degree of preference for each biobank profile.

Conjoint experiments are useful for testing hypotheses with large designs involving several dimensions/attributes with varying levels. This large design poses a problem for causal analysis because if we were to randomly assign a treatment per respondent, the combinations of attributes and levels forming an intervention/treatment for testing would be very large for an experiment. A conjoint experiment solves this issue by asking the respondents to rate multiple treatments simultaneously. It also randomly assigns levels within each attribute across treatments and individuals, so that we can estimate the effect of each attribute on an outcome variable of interest. The main assumptions were that: (1) there was a random assignment to all other level combinations (no attribute or level dropped while creating a combination); (2) ratings on the rating tasks were independent of one another; and (3) the ordering of biobank profiles within a choice set/task did not affect responses^24^.

Each respondent was asked to rate 16 biobank profiles (including all seven attributes) on a scale of 1–10. For this, the levels were randomized for each attribute to create various sets of biobank profiles. And thus, we had 2^7^ = 128 combinations. The respondents were asked to rate multiple biobank profiles simultaneously (4 questions each for a set of 4 profiles). Therefore, each person rated 16 biobank profiles in total. Since we surveyed 78 respondents and each ranked 16 options/combinations, we had rankings for 1248 biobank profiles in total. So, each unique combination got ranked 9.75 times (1248/128). Therefore, we had an adequately large sample to detect even small attribute-level effects and can be assured that every possible combination was ranked.

We tested several theoretical expectations, such as whether researchers would choose biobanks with low handling fees, had confidence in the quality biospecimens, well-characterized biospecimens, etc. We regressed respondents’ biobank profile ranking on their assignments to each level of each attribute, for each rating task. Our base empirical specification was a respondent fixed effect regression analysis, which accounted for any bias arising from unobserved differences in a respondent’s ranking scheme. We also had a choice set fixed effect to account for any bias arising from conducting the rankings as part of different choice sets. For instance, there could be more fatigue and disinterest while answering the fourth choice set involving the last four biobank profiles. Choice set fixed effect accounted for any such unaccounted differences in the ranking of choice sets across respondents. We included respondent characteristics such as education, years of experience, industry, etc. as controls in our second specification in the regression analysis. We clustered standard errors at an individual/respondent level to account for serial correlation in a person’s rating of various biobank profiles. All analyses were conducted using Stata 16.

## Results

This section describes the respondents involved in the conjoint experiment and the causal results of the incremental value of biobank attributes. We supplement the analysis by reporting the results by subgroups based on respondent characteristics, such as their past experience with biobanks, and individual characteristics, such as years of experience.

### Description of survey respondents/sample

Here we present descriptive statistics of the sample, followed by the results from the conjoint analysis. Table 2 shows the characteristics of the survey respondents. Approximately 77 percent of the respondents were researchers, either from academic institutions, health institutions, or bioanalysis research companies. Around 18 percent of the respondents were from diagnostic centers, and five percent worked in pharmaceutical companies. Respondents were from 25 countries, with the highest participation from countries in Africa (39 percent). The African continent has the highest underlying burden of endemic diseases in the world^26^. There is a new interest in establishing a biobanking network in Africa to ensure appropriate and timely diagnosis of diseases^27,28^. Since our survey-based experiment included testing of the value for bio-samples from key endemic sites, Therefore, we oversampled researchers from Africa to ensure that we get correct responses on the value of various biobank attributes. A majority of our sample (> 85 percent) had at least a postgraduate degree. Almost half of the respondents had purchased biological samples in the past, with 74 percent of respondents having used non-commercial biobanks. Out of those who had prior experience in purchasing biological samples, 65 percent were unable to access the requested biological samples in the past.

**Table 2:** Socio-economic demographic profile and past experience with bio-samples among survey respondents.

	Count	Percentage
Sector of employment		
Health research institutes/organizations	33	42.3
Biomedical researchers from universities	18	23.1
Diagnostic centers	14	17.9
Biomarker and bioanalysis research companies	9	11.5
Pharmaceutical companies	4	5.1
Geographical location		
Africa	30	39.4
Asia	13	17.1
Europe	18	23.6
North America	12	15.7
Oceania	1	1.3
South America	2	2.6
Years of experience		
Less than 1 year	1	1.32
1–5 years	15	19.74
5–10 years	18	23.68
More than 10 years	42	55.26
Education		
Undergraduate/Bachelor’s degree	10	13.16
Postgraduate/Master’s degree	24	31.58
Master of Philosophy (MPhil)	2	2.63
Doctor of Philosophy (PhD)	32	42.11
Medical degree or higher	8	10.53
Purchased a bio-sample in the past		
Yes	38	48.72
No	40	51.28
Type of biobank		
Commercial	10	26.32
Non-commercial/Non-profit	19	50
Both commercial and non-commercial	9	23.68
Unable to access a required bio-sample		
Yes	25	65.79
No	13	34.21
Time taken to obtain bio-sample		
Less than 1 month	4	10.53
1–3 months	16	42.11
3–6 months	12	31.58
6–12 months	5	13.16
More than 12 months	1	2.63

### Causal results on the value of biobanks

**Figure 1:**
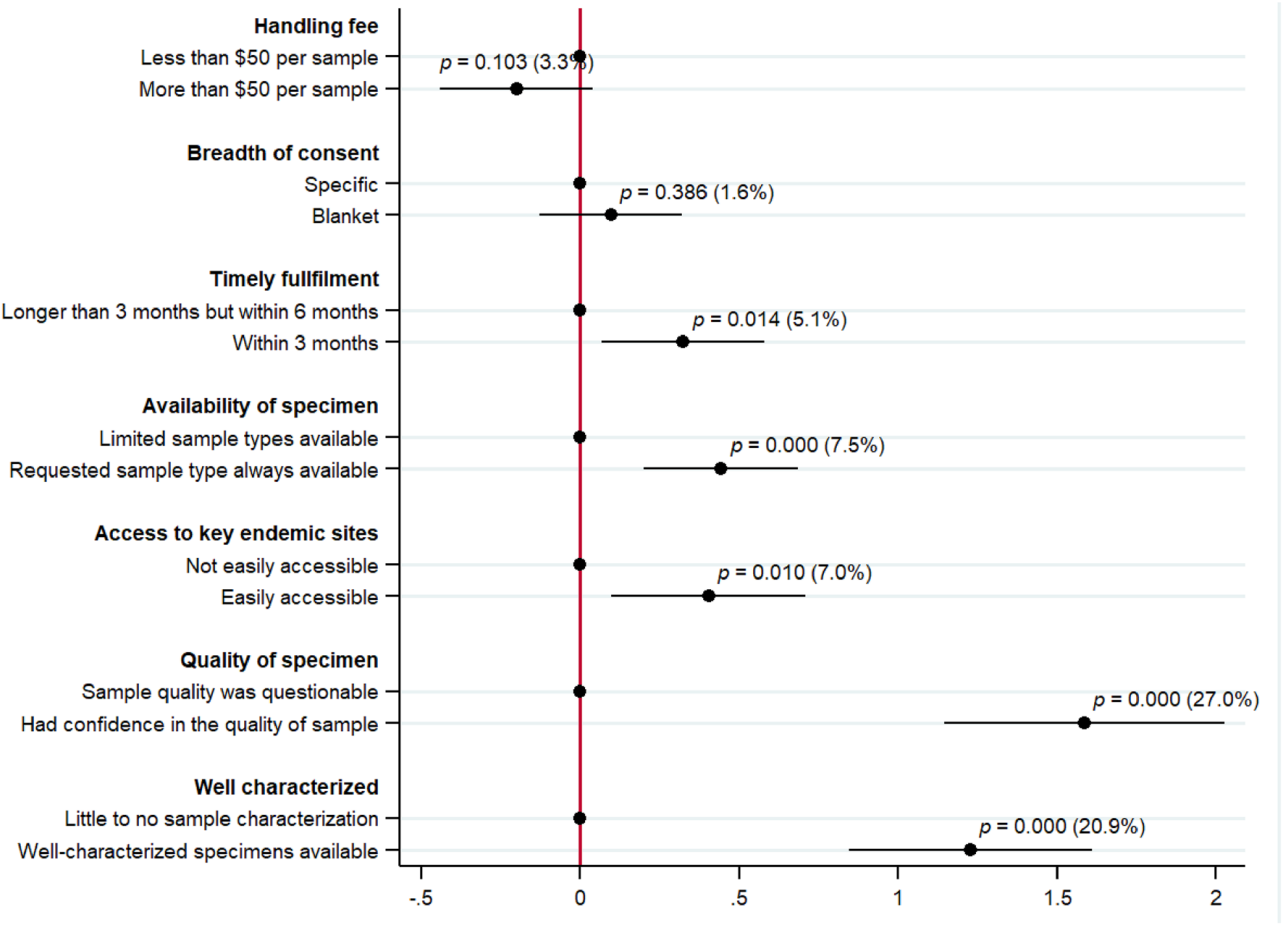
Incremental value of biobank attributes and their levels (in percentage). *Note* Regression results are presented in (circles) with 95 percent confidence intervals extending from each one. *P* values and percentage change compared to mean ranking are reported. Standard errors were clustered at the respondent/individual level.

We analyzed survey data from 78 respondents on 16 biobank profiles each. The biobank profiles were a random combination of seven attributes, each with two levels. Through this conjoint analysis, we aimed to understand the preferences of researchers on the incremental value of various attributes of biobanks. In Table 3, column 1, we report that respondents primarily preferred a biobank which provided them with confidence in the quality of biospecimens. We found an effect of 26 percent higher ranking for biobanks providing confidence in the quality of biospecimens over a mean ranking of 5.86 (see Fig. 1). This was significant at the one percent level (coefficient: 1.53, *p* value < 0.001). Our results were strengthened and remained significant (Table 3, col 2) with respondent-level controls (27 percent, coefficient: 1.59, *p* value < 0.001). All percentage changes from the mean ranking of 5.86 are reported in Fig. 1.

**Table 3:** Main regression results of the conjoint analysis.

	Respondent fixed effects (1)	Respondent fixed effects with controls (2)
High handling fee	− 0.21*	− 0.20
	(0.12)	(0.12)
Breadth of consent	0.14	0.10
	(0.12)	(0.11)
Timely fulfilment	0.30**	0.32**
	(0.13)	(0.13)
Availability of specimen	0.44***	0.44***
	(0.12)	(0.12)
Access to key endemic	0.41***	0.40**
	(0.15)	(0.15)
Quality of specimen	1.53***	1.59***
	(0.22)	(0.22)
Well characterized specimen	1.20***	1.23***
	(0.19)	(0.19)
Individual fixed effects	Yes	Yes
Choice set fixed effects	Yes	Yes
Other individual controls	No	Yes
Observations	1248	1216
Mean	5.86	5.86
R-Squared	0.51	0.52

Standard errors were clustered at the respondent/individual level and presented in paratheses.

***p < 0.01, **p < 0.05, *p < 0.1.

The second most desired attribute was the availability of well-characterized specimens (see Table 3, columns 1 and 2), with 20.4 percent to 20.9 percent higher ranking than the mean. This was significant at the one percent level. The third most desired attribute of biobanks for researchers was the availability of a variety of specimen types (versus limited availability of bio-samples), which was ranked 7.5 percent higher than the mean ranking (coefficient: 0.44, *p* value < 0.001). We also found that biobank consumers value easy access to biological samples from key endemic in-country sites. This fourth more preferred attribute was ranked seven percent higher than the mean ranking of 5.86, which was significant at the one percent level (See Table 3, columns 1 and 2) for both specifications with and without respondent characteristics controls. The fifth most valuable attribute to the respondents was the timely fulfilment of requests (i.e., receiving a specimen within three months of placing the request). This attribute was ranked about 5.1 percent more than the mean ranking and was significant at the five percent level. We did not find any significant preference for the biobank attributes related to the cost of acquiring the specimen (high versus low handling fee) and the breadth of consent (blanket versus specific) at the five percent level.

### Heterogeneous treatment effects on respondent characteristics

We also studied heterogeneous, or differential, treatment effects based on respondent characteristics such as research experience (more or less than 10 years) and past experience with biobanks. The value of biobanks may have differed for researchers based on their varying prior experience with biobanks. For instance, we expected researchers who were unable to access requested bio-samples in the past to provide higher ratings of the quality of specimens and access to key endemic sites; and researchers who did not receive requested bio-samples in a timely manner to provide higher ranking to biobanks with timely fulfilment of requests.

Almost half of our sample had prior experience purchasing biological samples, so we conducted subgroup analyses of respondents who: (1) had purchased from commercial versus non-commercial biobanks; (2) had experience receiving biological samples within three months of request versus more than three months; and (3) were able to access versus unable to access the requested bio-sample. The most valued attributes across these various subgroups remained the confidence in the quality of biospecimens and well-characterization of specimens (See Table 4, columns 1–10). These results are in line with the overall findings discussed earlier in Table 4. The quality of specimens was ranked highest by respondents who had purchased biological samples from a commercial biobank in the past (44.5 percent, coefficient: 2.69, *p* value < 0.001) and lowest by those unable to access requested bio-samples in the past (22.2 percent, coefficient: 1.32, *p* value < 0.001). Well-characterized specimens were ranked highest by those receiving bio-samples later than three months after placing a request (29.7 percent, coefficient: 1.6, *p* value < 0.001) and lowest by respondents with less than 10 years of experience (15.7 percent, coefficient: 0.88, *p* value < 0.001).

**Table 4:** Heterogeneous treatment effects on respondent characteristics.

	Ever purchased bio-sample	Never purchased bio-sample	Purchased from Commercial Biobanks	Non-commercial	Requested Bio-sample received in more than three months	Time taken lesser than three months	More than 10 years of experience	Experience of individual < 10 years	Unable to access requested bio-sample in the past	Able to access requested bio-sample in the past
	(1)	(2)	(3)	(4)	(5)	(6)	(7)	(8)	(9)	(10)
High handling fee	− 0.32**	− 0.11	− 0.20	− 0.33	− 0.08	− 0.63**	− 0.18	− 0.23*	− 0.40*	− 0.10
	(0.15)	(0.18)	(0.19)	(0.22)	(0.20)	(0.24)	(0.19)	(0.13)	(0.21)	(0.23)
Breadth of consent	0.19	0.04	0.17	0.16	0.12	0.29	0.18	− 0.02	0.20	0.22
	(0.16)	(0.16)	(0.28)	(0.19)	(0.23)	(0.18)	(0.16)	(0.16)	(0.22)	(0.24)
Timely fulfilment	0.36*	0.29*	0.93**	0.19	0.45	0.17	0.23	0.42**	0.42	0.20
	(0.21)	(0.15)	(0.34)	(0.25)	(0.30)	(0.30)	(0.19)	(0.16)	(0.29)	(0.30)
Availability of specimen	0.37*	0.48**	0.44	0.35	0.48*	0.26	0.49***	0.35*	0.44*	0.30
	(0.18)	(0.18)	(0.36)	(0.23)	(0.27)	(0.23)	(0.16)	(0.17)	(0.25)	(0.30)
Access to key endemic	0.68**	0.14	0.51*	0.71**	0.67*	0.73*	0.51**	0.25	0.76*	0.49*
	(0.25)	(0.16)	(0.26)	(0.34)	(0.37)	(0.38)	(0.22)	(0.21)	(0.37)	(0.26)
Quality of specimen	1.57***	1.59***	2.69***	1.17***	1.60***	1.62***	1.35***	1.89***	1.32***	2.01***
	(0.30)	(0.32)	(0.62)	(0.30)	(0.46)	(0.39)	(0.28)	(0.35)	(0.36)	(0.53)
Well characterized	1.41***	1.04***	1.27**	1.52***	1.60***	1.24**	1.53***	0.88***	1.59***	1.14**
	(0.31)	(0.23)	(0.42)	(0.40)	(0.37)	(0.52)	(0.31)	(0.17)	(0.43)	(0.40)
Observations	592	624	160	432	320	272	672	544	384	208
R-squared	0.56	0.50	0.53	0.59	0.45	0.65	0.48	0.58	0.55	0.60
Mean	5.85	5.86	6.04	5.79	5.95	5.76	5.93	5.77	6.09	5.59

Standard errors were clustered at the respondent/individual level and presented in paratheses.

***p < 0.01, **p < 0.05, *p < 0.1.

The other attributes such as availability of a variety of specimens, access to key endemic sites, and time taken to fulfil requests were valued significantly for only certain subgroups of respondents (see Table 4, columns 1–10). Availability of a variety of specimen types (see Table 4), was ranked significantly (*p* < 0.05) higher only for respondents who had never purchased a biological sample (8.1 percent, coefficient: 0.48, *p* value < 0.05) and respondents with more than 10 years of experience (8.1 percent, coefficient: 0.49, *p* value < 0.05). Similarly, the fourth most desired attribute of access to key endemic in-country sites was significant only for those respondents who had previously purchased a bio-sample (11.6 percent, coefficient: 0.68, *p* value < 0.05), purchased from non-commercial biobanks (12.2 percent, coefficient: 0.71, *p* value < 0.05), and had more than 10 years of experience (8.4 percent, coefficient: 0.51, *p* value < 0.05). The fifth desired attribute of timely fulfilment of biological sample requests was ranked significantly higher than the mean ranking by those with experience purchasing biological samples from commercial biobanks (15.4 percent, coefficient: 0.93, *p* value < 0.05) and with less than 10 years of research experience (7.5 percent, coefficient: 0.42, *p* value < 0.05).

The cost of acquiring the bio-sample or the handling fee was significant for several groups of respondents in the heterogeneous treatment effect. According to the results reported in Table 4, columns 1 and 6, respondents who had purchased a biological sample in the past (− 5.5 percent, coefficient: − 0.32, *p* value < 0.05) and received a bio-sample within three months of requesting it (− 10.9 percent, coefficient: − 0.63, *p* value < 0.05) were sensitive to high handling fees charged by biobanks. They ranked the biobank attribute of high handling fees significantly lower (by 0.32–0.63 percentage points) than the mean ranking of 5.85. The attribute of the breadth of consent was not significant in the overall or subgroup analysis.

## Discussion

The COVID-19 pandemic has highlighted the value of biological samples for developing effective vaccines in an urgent manner to deal with outbreaks of infectious diseases^29–31^. Biobanks play an essential role in researching various factors that may affect human health such as long-term consequences of COVID-19, detection of major human malaria species, rapid and consistent protein identification, etc.^32–34^. Therefore, timely access, availability of low-cost, well-characterization and confidence in the quality of the biospecimens is required for future health care research.

The results of the conjoint experiment conducted in this study reveal that different characteristics of biobanks can have a significant impact on researchers’ preferences of biobanks. Experts in the biomedical and health research especially value biobanks offering biospecimens with confidence in the quality and well-characterization. Apart from these two most prominently ranked features of biobanks, some other significant features valued by respondents who had purchased a bio-sample in the past were access to key endemic sites and low handling fees of bio-samples. In the overall sample, availability of a variety of specimens and timely fulfilment of requests also appeared to be significantly valued features of biobanks.

This is a novel study as there is no prior causal evidence on value of biobank attributes to researchers. However, this study is not one without limitations. Our main limitation is that the study is based on self-reported biobank profile ratings. The degree to which these ratings are associated with uptake behavior for specific biobanks is an open question. Second, similar to most conjoint analysis or preference studies^35,36^, the incremental value of attributes elicited in our study is limited to the attributes and levels presented for ranking. It is possible that some attributes and levels desired by respondents were not included. Despite these limitations, the study has many strengths and successfully finds causal effects of multiple treatment (or biobank attributes) simultaneously.

Our results have important policy implications for the public health research domain and both short-term and long-term health care research. Existing biobanks can strengthen their capacity based on the findings of this research to increase their value and demand in the market. Our findings indicate that biobanks can perform better as a research tool if they focus on providing biospecimens with confidence in the quality and well-characterization. There have also been concerns about commercialization of biobanks due to funding challenges and discussions about potential misuse of blanket consent of participants. We do find researchers who had prior experience acquiring bio-samples to be more price sensitive with regard to handling fees and perhaps to other costs too, but we do not find blanket consent of participants to be significantly valued by researchers.

## Ethics

Since this is a global study (with jurisdiction issues), the IRB board Tech4Health IEC exempted the study.

## Data Availability

The raw data collected on the value of biobanks is protected and not available to maintain the anonymity of the participants. The de-identified processed data can be made available by making a request to the corresponding author DB.
